# Research Focus in Palliative Care

**DOI:** 10.4103/0973-1075.76231

**Published:** 2011-01

**Authors:** Bidhu K Mohanti

**Affiliations:** Department of Surgical Oncology, IRCH All India Institute of Medical sciences, New Delhi, India

**Keywords:** Palliative care, Research, India

## Abstract

*This brief article on pre-conference CME topic ‘How to plan Research in Palliative Care’ is aimed to provide an overview of the background, concept, domains, present research activities and the future prospect for research opportunities*. Advances in Palliative Care are made with a focus to address the quality of medical practice and ‘quality of death’, in those patients who have advanced stage diseases where cure may or may not be possible. The issues which can improve the palliative care delivery and the areas where evidence of practice is still weak can be identified by forming network and collaborative groups for the application of study and research methods in India.

This brief article on pre-conference CME topic “How to plan Research in Palliative Care” is aimed to provide an overview of the background, concept, domains, present research activities and the future prospect for research opportunities.

*Bidhu K Mohanti*

Research in health care has always fascinated the mankind. The benefits of penicillin, safety of surgery under anesthesia, global eradication of small pox by vaccination, discovery of X-rays, and our recently unfolding human genome project are some of these examples of medical research. Advances in palliative care are made with a focus to address the quality of medical practice and “quality of death” in those patients who have advanced stage diseases where cure may or may not be possible.

The recent World Health Organization (WHO, 2008) definition of palliative care is set as “an approach that improves the quality of life of patients and their families facing the problems associated with life-threatening illness, through the prevention and relief of suffering by means of early identification and impeccable assessment and treatment of pain and other problems, physical, psychosocial and spiritual.”

Research focus in palliative care is to be aimed to cover all the above aspects mentioned in the WHO definition. Good medical practice requires evidence of effectiveness. It has become clear that developments in palliative care are to be considered as efforts to address deficits in care, strive for further improvements and justly apportion the limited resources.[1] Sustainable and quality research in India will be possible by establishing a network of individuals – doctors, nurses, paramedics, other professionals, institutions and organizations, including commercial establishments who have a stake in the palliative care practice. The issues that can improve the palliative care delivery and the areas where evidence of practice is still weak can be identified by forming network and collaborative groups for the application of study and research methods in India.

## A BRIEF HISTORY

Care of the terminally ill, infirm and elderly individuals has been a key part of many societies in the world. Since the 4^th^ century, rest house, sarai, sanatorium and hot springs were developed as special places to attend to their needs. The diagnosis of cancer and understanding of its incurability in a majority of patients, in the first half of 20^th^ century, appeared as new challenges to the medical community. It was gradually realized that the needs of terminal patients with advanced malignant disease were not met by the then prevailing specialist or non-specialist health systems. The pioneering works of Dame Cicely Saunders in the United Kingdom drew the attention of the medical community and the public to the evolution of palliative care in the 1960s. From the 1980s, rapid progress was made in developing palliative care as a discipline in the health care delivery.

In the 21^st^ century, many countries have recognized that palliative care is a medical specialty, with a distinct emphasis on education, training, clinical practice skills, manpower, structure and settings.[2]

## THE PROGRESS

The basic concepts of palliative care were developed between 1960 and 1990, mainly in the field of oncology. Traditional medical practices showed the deficits in dealing with cancer patients and their families when the disease was progressive and incurable. Patients faced with pain, distressing physical symptoms and fear of dying required a continuum of medical care. A holistic approach was evolved toward communication with the patient and family, place of care between hospital, hospice and home, assessment of the patient’s symptoms, medical interventions and therapy to relieve the symptoms and addressing the emotion and anxieties. These have been the key domains of activities in palliative care. In the early years, pain was recognized as a dominant factor affecting the patient’s quality of life, and pain relief approaches were meticulously studied by Dame Cicely Saunders. Her narrative about the first encounter with a terminal cancer patient, David Tasma, who told her “*I will be a window in your Home*” remains the classic documentation of scientific concept in palliative care.[3] The initiatives to implement and develop palliative care were taken up in the 1960s and `70s by the voluntary sector. Hospices, palliative care clinics and day care facilities were established across the UK, Europe, USA, Canada, etc. The recognition of palliative care as an integral part of cancer control policy and the guidelines for morphine in cancer pain relief by the WHO were salutary efforts made in the 1990s. These two factors propelled the national policies of many countries to implement palliative care in the last 20 years. In India, the earliest facilities to deliver palliative care within cancer centers were established in some places like Ahmedabad, Bangalore, Mumbai, Trivandrum and Delhi in the late 1980s and the early 1990s.The Indian Association of Palliative Care (IAPC) was formed in 1994, at the venue of a conference on palliative care held at Varanasi. The IAPC was constituted by a small group of like-minded individuals, in consultation with the WHO and the Government of India, with a mission “*to promote affordable and quality palliative care across the country*.” The journal of IAPC was started later and the Indian Journal of Palliative Care (IJPC) is now in its 16^th^ year of publication (www.palliativecare.in).[4]

## THE PRESENT CHALLENGES

The reasons to conduct research in palliative care are that there at many levels within our health system.

Fifty percent of the patients with cancer are not cured of their disease. However, with improved treatments, even those with advanced stage may live for many years. Providing palliative care for those who are incurable, and for patients in advanced stage concurrent with anticancer therapy, has been proposed to improve their quality of life.[5]

The limited availability of palliative care service structures within a hospital or outside make it difficult for the patients and their families to go through the terminal phase of disease and dying. This is further compounded by the lack of an adequate number of health professionals trained to deliver the palliative and end-of-life care. These infrastructure and personnel deficits are observed even recently in many developed countries like the USA and Germany.[6,7]

The physicians and nurses who deliver community health care are not educated and trained in the domains of palliative care, whereas a large part of managing the patients with life-threatening diseases like cancer and other end-stage conditions require medical attention nearer to their homes.[7]

Availability and procurement of morphine, an essential drug in palliative care, is often an obstacle in many countries. Although relief from pain, distressing symptoms and dignity in death are considered as rights of patients with advanced cancer and other end-stage diseases, recent reviews and observational studies describe considerable dissatisfaction, indicating that there are still opportunities for improvement.[8]

## PRESENT ACTIVITIES

Research approaches are required to study the various issues that should be improved or overcome in India. The present works being done in other parts of the world can provide helpful information and guidance. The European Association for Palliative Care (www.eapcnet.eu), Palliative Care Research Society (www.pcrs.org.uk), National Cancer Research Institute (www.ncri.org.uk) in the UK; the NIH, Institute of Medicine and National Palliative Care Research Centre (www.npcrc.org) in the USA; Palliative Care Research Program under National Health and Medical Research Council, Australia (www.nhmrc.gov.au); and Canadian Hospice Palliative Care Association (www.chpca.net/researcher_registry) are some of the organizations in the forefront of palliative care research.

As seen from the websites of these organizations and the site of ClinicalTrials.gov, some examples of research activities being conducted presently are:

- basic and translational pharmacogenetics opioid study,
- therapeutic/clinical-cancer cachexia intervention study,
- family-focused group therapy, psychosocial support intervention,
- Sativex^R^ in advanced cancer-related pain,
- palliative chemotherapy in pancreatic cancer,
- stent with or without radiotherapy in advanced esophageal and biliary malignancies,
- study on palliative care service models and their effectiveness.

## OPPORTUNITIES FOR RESEARCH IN INDIA

A major percentage of advanced and end-stage patients with cancer, HIV/AIDS and other life-limiting diseases are seen in the developing countries of Asia, Africa and South America. However, palliative care is still unavailable to most of these patients. Effective and resource-appropriate palliative care models are lacking in these countries. Research from developing countries is therefore needed to develop, implement and monitor the delivery of palliative care in ways that are feasible in resource-poor settings and acceptable to local populations.[9]

Indian initiatives for research in palliative care have been largely shouldered by individuals within medical institutions or, occasionally, by a group (Harris T, 2003).[10]

At present, the IAPC is in its 17^th^ year, and there are more than 150 centers actively engaged in palliative care delivery. The majority of the doctors, nurses and paramedical professionals working in these centers have rendered exemplary services to deliver palliative care to cancer and other terminally ill patients, raise awareness about palliative care practice and educate others.[11] The existing strength in India, i.e. IAPC, the journal IJPC, palliative care facilities, experts, and professionals in this field should be jointly utilized to mount coordinated research activities.

Evidence of advancement in palliative care outcomes are studied in many countries where the specialty and non-specialty facilities are well structured. The health system evaluation experts and palliative care researchers have identified the key domains that represent the success.[6] These are outlined in Table 1.

**Table 1:** The national consensus project domains, USA[6]

Domains	Description
Domain 1	Structure and process of care
Domain 2	Physical aspects of care
Domain 3	Psychological and psychiatric aspects of care
Domain 4	Social aspects of care
Domain 5	Spiritual, religious and existential aspects of care
Domain 6	Cultural aspects of care
Domain 7	The imminently dying patient
Domain 8	Ethical and legal aspects of care

The palliative care professionals, centers and academic institutions in India can identify the areas for research within these domains. Patients in palliative care are in need of multi-dimensional approaches toward disease condition, physical symptoms, psychosocial support, family characteristics and terminal/dying state. Hence, the palliative care professionals often have to seek interdisciplinary management and should look for collaborative research with other specialties, e.g. palliative radiotherapy/chemotherapy, physical rehabilitation, etc.[12]

Research areas in palliative care can be similar to other disciplines of medicine, encompassing the basic, translational and clinical levels. These can be genomic assay for opioid resistance and comparison of drugs and interventions in relief of a symptom like dyspnoea or anxiety. At the same time, the mix of in-patient, home care, hospital clinic and day care settings during the delivery of palliative care can be utilized to address the economic impact or how quality of life of the patients and the family carers differ according to the care environment.

It is often argued that patient-centered intervention and therapeutic effectiveness are ideally observed through prospective randomized clinical trials (RCTs). There are methodological issues to conduct RCTs in palliative and end-of-life care, such as difficulties with recruitment and attrition, inability to observe for sufficient time period to detect small differences and the ethical issue to compare practices or interventions in an ill and vulnerable patient population. To overcome these inordinate difficulties, the researchers in palliative care may have to look for alternatives. Well-designed and meticulously conducted observational studies, cross-sectional surveys and prospective documentations can minimize the bias and provide insightful results equivalent to RCTs (Wee B, 2008).[13]

Palliative care delivery in India faces the hurdle of resources. Even the western models are being questioned. Are the hospices meant for “luxurious dying”? Is home care meant for those “who can be reached easily”? Hence, there is scope for improvements in distribution of palliative care settings between hospital, home and hospice, something more practical, accessible and less expensive in India!

We can draw lessons from a recent study on how quality research in palliative care is improving the survival in advanced lung cancer patients. It has come to highlight the hitherto unknown benefits of relieving the disease-related symptoms while the patient is receiving anticancer therapy.[14]

In summary, there is good scope for developing a research culture in the Indian palliative care scenario. It is imperative to form collaborative groups, identify issues and areas for research, design resource-appropriate studies, encourage researchers through grants and seek public and private funds for conducting short-term and long-term researches as trials and studies. These activities will create an atmosphere of ideas and evidences in India, and will develop networking both within the country and outside with other countries of the world.

While looking at different sources to prepare this note, I came chanced upon reading the one-liner motto of the National Palliative Care Research Center, New York, USA (on its home page, www.npcrc.org), which says:

“*Without research, palliative care is an art, not a science*”
